# The therapeutic potential of the cerebellum in schizophrenia

**DOI:** 10.3389/fnsys.2014.00163

**Published:** 2014-09-15

**Authors:** Krystal L. Parker, Nandakumar S. Narayanan, Nancy C. Andreasen

**Affiliations:** ^1^Department of Neurology, University of IowaIowa City, IA, USA; ^2^Department of Psychiatry, University of IowaIowa City, IA, USA

**Keywords:** schizophrenia, cerebellum, anterior cingulate, cognitive symptoms, optogenetic stimulation

## Abstract

The cognitive role of the cerebellum is critically tied to its distributed connections throughout the brain. Accumulating evidence from anatomical, structural and functional imaging, and lesion studies advocate a cognitive network involving indirect connections between the cerebellum and non-motor areas in the prefrontal cortex. Cerebellar stimulation dynamically influences activity in several regions of the frontal cortex and effectively improves cognition in schizophrenia. In this manuscript, we summarize current literature on the cingulocerebellar circuit and we introduce a method to interrogate this circuit combining opotogenetics, neuropharmacology, and electrophysiology in awake-behaving animals while minimizing incidental stimulation of neighboring cerebellar nuclei. We propose the novel hypothesis that optogenetic cerebellar stimulation can restore aberrant frontal activity and rescue impaired cognition in schizophrenia. We focus on how a known cognitive region in the frontal cortex, the anterior cingulate, is influenced by the cerebellum. This circuit is of particular interest because it has been confirmed using tracing studies, neuroimaging reveals its role in cognitive tasks, it is conserved from rodents to humans, and diseases such as schizophrenia and autism appear in its aberrancy. Novel tract tracing results presented here provide support for how these two areas communicate. The primary pathway involves a disynaptic connection between the cerebellar dentate nuclei (DN) and the anterior cingulate cortex. Secondarily, the pathway from cerebellar fastigial nuclei (FN) to the ventral tegmental area, which supplies dopamine to the prefrontal cortex, may play a role as schizophrenia characteristically involves dopamine deficiencies. We hope that the hypothesis described here will inspire new therapeutic strategies targeting currently untreatable cognitive impairments in schizophrenia.

## Introduction

The purpose of this theory and hypothesis manuscript is two fold. First, we review the role of the cerebellum in cognition and schizophrenia, the anatomy of cerebellar projections to frontal cortex, and the available evidence indicating that the cerebellum influences the frontal cortex. Second, we introduce the novel hypothesis that optogenetic cerebellar stimulation may ameliorate cognitive symptoms of schizophrenia by normalizing frontal activity, and we propose testable animal and translational experiments to probe this hypothesis. Studies have shown that connections exist between the frontal cortex and cerebellum and that cerebellar stimulation improves cognitive symptoms of schizophrenia; to our knowledge, this manuscript is the first to propose a method to pharmacologically disrupt frontal function in rodents to mimic abnormalities in schizophrenia, document cognitive dysfunction similar to that reported in schizophrenia, and use cerebellar stimulation to restore frontal activity and rescue cognition. Specifically, we will evaluate the therapeutic potential of the cerebellum by introducing a technique to interrogate cerebellar connections to the frontal cortex using combined opotogenetics, neuropharmacology, and electrophysiology in awake-behaving animals while minimizing incidental stimulation of neighboring cerebellar nuclei.

The cerebellum is a critical component in tasks of both motor and cognitive origin. Thalamic connections between the cerebellum and frontal cortex create the potential for the cerebellum to powerfully influence cognition. In schizophrenia, there are abnormalities in all three regions, which led to the hypothesis that a distributed network involving the cerebellum contributes to cognitive deficits. We, and many of our colleagues, have been investigating this circuit for almost two decades (Leiner et al., 1994; Andreasen et al., 1998; Schmahmann, 1998, 2010; Andreasen and Pierson, 2008). Although studies of functional neuroimaging, anatomy, positron emission tomography (PET), and structural and functional magnetic resonance imaging are extensive, it is unclear how this circuitry is influenced by schizophrenia (Alphs, 2006). The advent of molecular tools and imaging technology such as tractography and diffusion tensor imaging (DTI) that can be used in psychiatric disorders have provided additional clarity to this issue (White et al., 2008).

Cognitive impairments in schizophrenia including executive dysfunctions in working and episodic memory, (Andreasen et al., 1999; Ragland et al., 2009), attention, reasoning, and timing (Carroll et al., 2009), remain untreatable (Alphs, 2006). These impairments have been described as cognitive dysmetrias, or disruptions in the synchronous coordination of cognitive capacities (Andreasen et al., 1998; Schmahmann, 1998). Recently, Demirtas-Tatlidede et al. reported that cerebellar vermal theta burst stimulation (TBS) has been effective at relieving some cognitive symptoms in treatment-resistant schizophrenia patients (Demirtas-Tatlidede et al., 2010). Specifically, eight schizophrenia patients were evaluated using comprehensive neuropsychological testing, Positive and Negative symptoms subscale (PANSS), Clinical Global Impression (CGI), Calgary Depression Scale for schizophrenics (CDSS), Profile of Mood States (POMS), and Visual Analogue Mood Scale (VAS) for dimensions of mood (Happiness, Sadness, Calmness, Anxiety, Wellbeing, Anger, Self-confidence, Fear, Alertness, and Energy). Subjects received cerebellar vermal TBS twice daily on 5 consecutive days for a total of 10 stimulation sessions. Baseline ratings were compared with ratings immediately following stimulation and 1 week later. The results showed significant improvement in negative symptoms reported by the PANSS both following treatment and at follow-up, and there was a significant elevation in mood. Cognition also improved as the subject had fewer working memory omissions with no worsening of other measures.

The mechanism underlying the efficacy of cerebellar stimulation in schizophrenia is unknown. Studies have shown that electrically stimulating the cerebellum produces downstream changes in the prefrontal cortex and anterior cingulate cortex (Mittleman et al., 2008; Rogers et al., 2011, 2013; Watson et al., 2014). Connections between the cerebellum and anterior cingulate, comprising a cingulocerebellar circuit, are of particular interest because they have been confirmed to exist by tracing studies, they are conserved between species including humans, primates, and rodents, and structural and functional abnormalities are present in schizophrenia. Here, we present empirical evidence that (1) the deep cerebellar nuclei and anterior cingulate cortex are disynaptically connected via two separate pathways (the thalamus and the ventral tegemental area—VTA) allowing the cerebellum access to the frontal cortex; and (2) the cerebellum may modulate frontal neuron ensembles. This cerebellar modulatory mechanism has the potential to be harnessed to rescue abnormalities in the anterior cingulate, typically associated with schizophrenia. The mechanism underlying the efficacy of cerebellar stimulation needs to be illuminated if cerebellar stimulation is to become a therapeutic treatment strategy for the negative and cognitive symptoms of schizophrenia (Demirtas-Tatlidede et al., 2013).

## Cingulocerebellar interactions in schizophrenia

### Functional neuroimaging

**Figure 1:**
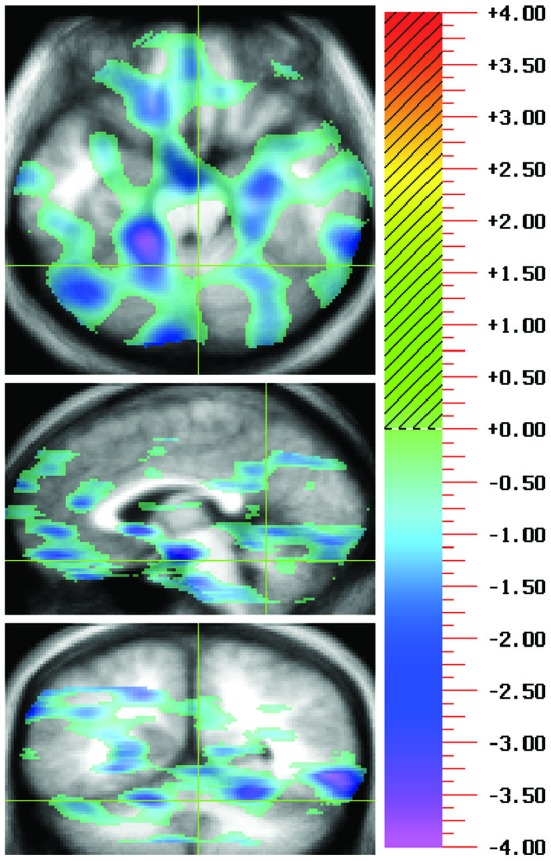
**During positron emissions tomography (PET) imaging of a task typically thought to be cerebellum dependent, eyeblink conditioning, hypofunction was revealed in the cingulocerebellar circuit**. A double subtraction method where the baseline pseudoconditioning phase of unpaired tones and airpuffs was first subtracted and then the rCBF for patients with schizophrenia was subtracted from that of controls, show negative peaks indicating patients with schizophrenia have less rCBF than controls. This representative image shows hypofunction of the anterior cingulate (Talairach coordinates: −1, 30, 14) and cerebellar lobules IV/V, and IX (Talairach coordinates: −3, −67, −17) in patients with schizophrenia in comparison to healthy controls. For each illustration there are three orthogonal views per row with transaxial on the top, sagittal in the middle, and coronal on the bottom. Green crosshairs are used to show the location of the slice. Images follow radiological convention and show location as if facing the patient where the left side of the image represents the patient’s right side. The statistical maps of the PET, showing the regions where the two groups differed significantly at the 0.005 level, are superimposed on a composite magnetic resonance image (MRI) derived by averaging the MR scans from the subjects. Regions in red/yellow tones indicate positive peaks (greater activation in patients than in controls) and regions with blue/purple indicate negative peaks (less activity in patients than controls). The statistical results are portrayed using the value of the associated *t*-statistic, which is shown on the color bar on the right. The images are referred to as “*t* maps” showing all voxels in the image which exceed a display threshold.

Cognitive dysmetria has been probed in patients with schizophrenia (Andreasen and Pierson, 2008) concurrently undergoing neuroimaging using tasks such as recall of complex narratives (Andreasen et al., 1995a), episodic memory (Andreasen et al., 1999; Ragland et al., 2009), memory for word lists (Andreasen et al., 1995b; Paradiso et al., 1997; Crespo-Facorro et al., 1999), recognition memory (Crespo-Facorro et al., 2001), dichotic listening (O’Leary et al., 1996), eyeblink conditioning (Parker et al., 2013b; Figure 1), and timing (Volz et al., 2001). These data consistently indicate lower blood flow in the cerebellum and frontal cortex (Andreasen et al., 1997) including medial frontal regions such as the anterior cingulate (Adams and David, 2007).

The correlation of these cognitive deficits with abnormal regional cerebral blood flow, led to the mechanistic explanation that abnormalities in a distributed cerebellar neural circuit may underlie cognitive impairments in schizophrenia (Andreasen et al., 1998). Aberrant connections from the cerebellum to the cerebral cortex may influence misconnections between percepts and their meanings, in turn causing errors in perceptual binding and misinterpretations of many kinds (e.g., delusions, hallucinations); they may also lead to inefficient or inaccurate information processing, forming the basis for the multiple types of cognitive impairments observed in schizophrenia (Andreasen et al., 1998; Wiser et al., 1998; Schmahmann, 2004).

The precise networks that are dysfunctional in schizophrenia remain elusive. Of particular interest is the anterior cingulate (Brodmann Areas 24/32) for its involvement in normal cognition and executive functioning including working memory, attention, emotional processing, response inhibition, performance monitoring, and timing (Devinsky et al., 1995; Narayanan et al., 2005, 2013; Picton et al., 2006; Cavanagh et al., 2009; Prabhakaran et al., 2011; Gasquoine, 2013). In addition, the anterior cingulate is consistently hypoactive in schizophrenia during cognitive tasks (Adams and David, 2007) such as random number generation (Artiges et al., 2000; Meyer-Lindenberg et al., 2001), error detection (Carter et al., 2001), monitoring self-performance (Carter et al., 1998, 2001), Stoop Tasks (Yücel et al., 2002; Minzenberg et al., 2009), continuous performance (Honey et al., 2005), and timing (Volz et al., 2001). Many of these studies found correlated cerebellar functional abnormalities (Honey et al., 2005; Koziol et al., 2013). These data provide evidence that cognitive impairments in schizophrenia may result from abnormalities in the cingulocerebellar circuit.

### Structural abnormalities

In addition to hypofunction, studies report structural abnormalities in the anterior cingulate, thalamus, and cerebellum in schizophrenia. Specifically, voxel based morphometry (Mouchet-Mages et al., 2011) and DTI (Wiser et al., 1998) reveal abnormalities in white matter connectivity between the nodes of the cingulocerebellar circuit in schizophrenia. The presence of structural abnormalities in these structures supports abnormalities in the distributed cerebellar network.

The cingulum bundle, including the anterior cingulate, has consistently shown abnormalities in schizophrenia (White et al., 2008). Patients with schizophrenia have an overall reduction of gray matter in the anterior cingulate (Brodmann Area 32) (Glahn et al., 2008; Takayanagi et al., 2013; Salgado-Pineda et al., 2014). Post mortem studies revealed a reduction in laminar thickness in the anterior cingulate (dorsal and subcullosal regions) (Fornito et al., 2009). Mitelman et al. report that schizophrenia patients with better outcomes have correspondingly higher fractional anisotropy (FA) than normal in frontal white matter areas including bilateral cingulate gyri; emphasizing the integral role of the anterior cingulate, he proposed that this increased FA in cingulate white matter may serve a neuroprotective role as indicated by a better outcome for patients (Mitelman et al., 2006). Our data support this hypothesis, as our patients with larger anterior cingulate volumes reported greater psychotic symptom improvement overtime (McCormick et al., 2005). In addition, functional and structural imaging data found convergent abnormalities in the medial frontal cortex including the anterior cingulate (Fornito et al., 2009; Pomarol-Clotet et al., 2010).

Structural abnormalities have also been revealed in thalamic nuclei and their projections in schizophrenia (Andreasen et al., 1994; Magnotta et al., 2008). Specifically, the thalamus has been shown to be reduced in size in schizophrenia (Andreasen et al., 1994; Buschman and Miller, 2007). In addition, FA was decreased in the internal capsule connecting the thalamus to the anterior cingulate (Oh et al., 2009). DTI reveals reduced FA in the white matter fiber tracts located between the thalamus and cerebellum in patients with schizophrenia compared to normal controls. Specifically, there was reduced FA within the superior cerebellar peduncle but not along the tract from the cerebellum to the thalamus (Magnotta et al., 2008). Little is known about whether the thalamus is essentially involved in schizophrenia or whether it is simply a convergence point and relay station for signals from other parts of the brain (Saalmann and Kastner, 2011). However, medial thalamic nuclei have been shown to project to the anterior cingulate cortex and inactivating these projections impairs working memory (Hsu and Shyu, 1997; Parnaudeau et al., 2013).

We have also reported that our schizophrenia patients have decreased overall cerebellar volume and, more specifically, decreased volume of the anterior lobe of the cerebellar vermis. Decreased cerebellar volume correlated with longer duration of psychotic and negative symptoms, and greater psychosocial impairment (Nopoulos et al., 1999; Wassink et al., 1999). Post mortem morphometric analyses of patients with schizophrenia confirm decreased anterior cerebellar vermis volume (Weinberger et al., 1980). A reduction of gray matter in Crus I and II of lobule VII has also been reported (Kühn et al., 2012). There may also be reduced FA in the vermis and middle cerebellar peduncles of schizophrenia patients (Okugawa et al., 2003, 2005).

Human lesion studies provide support for cingulocerebellar involvement in cognition. Although cerebellar lesions predominantly reveal motor impairments (Groiss and Ugawa, 2013), patients also suffer from comorbid cognitive impairments (Gottwald et al., 2004; Grimaldi and Manto, 2012). These dysfunctions include impaired timing, attention, memory, and language, all of which classically rely on the frontal lobes (Akshoomoff and Courchesne, 1992; Fiez et al., 1992; Grafman et al., 1992; Courchesne et al., 1994; Stoodley and Schmahmann, 2009). Schmahmann’s cerebellar cognitive affective syndrome has shown that human cerebellar lesions cause cognitive impairments (Schmahmann, 2004). Inducing transient impairments using repetitive transcranial cerebellar stimulation has recently been shown to impair cognitive tasks such as language, emotion, learning, memory, perception, and timing (Fierro et al., 2007; Koch et al., 2007; Oliveri et al., 2007; Grube et al., 2010; Bijsterbosch et al., 2011; Boehringer et al., 2013; Tomlinson et al., 2013; Grimaldi et al., 2014). Similar deficits are reported following lesions to the anterior cingulate (Devinsky et al., 1995). Specifically, timing on the go-nogo task, which probes response inhibition, has been shown to be impaired (Picton et al., 2006).

### Anatomical connections

**Figure 2:**
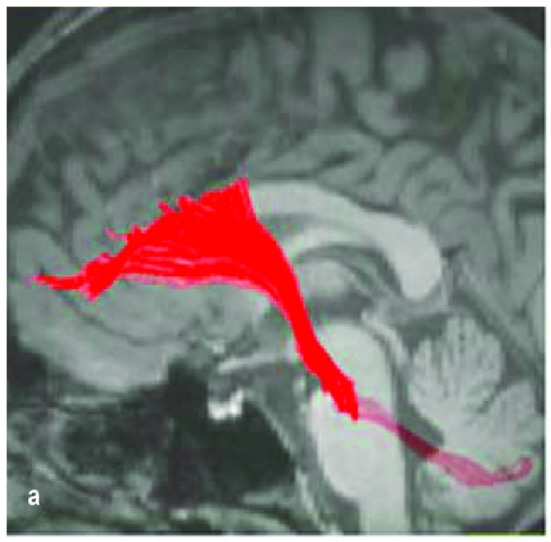
**Fronto-ponto-cerebellar tractography reconstructed on a 3D T1- weighted image (Kamali et al., 2010)**.

Neuronal tract-tracing has made giant strides to link the cerebellum to prefrontal networks, but many have focused exclusively on the dorsal lateral prefrontal cortex, which rodents lack (Uylings et al., 2003). The dorsal lateral prefrontal cortex (Brodmann Areas 9/46) has been the subject of extensive tracing studies in primates, which establish a “closed-loop” linking cerebellar nuclei (specifically the dentate) and the dorsal lateral prefrontal cortex via the contralateral thalamus. Retrograde tracer infusions in the dorsolateral prefrontal cortex and DTI reveal projections back to the deep cerebellar nuclei and cerebellar cortex via the pontine nuclei (PN; Middleton and Strick, 2001; Kamali et al., 2010; Schulz et al., 2014; Figure 2). These studies establish the potential for cerebellar-prefrontal interactions in primates but additional research is necessary (Uylings et al., 2003).

As we have argued, the anterior cingulate cortex is essential for normal cognition and shows impairments in schizophrenia. In humans, the anterior cingulate is also known as Brodmann areas 24/32 or the prelimbic cortices. Although homogenous in rodents, nomenclature is inconsistent. In rodents, the anterior cingulate cortex (Cg3/prelimbic) is commonly known as the medial prefrontal cortex and is described based on four cytoarchitecturally unique regions, Cg1, Cg2, Cg3/prelimbic, and infralimbic (Bostan et al., 2013; Vogt et al., 2013; Vogt and Paxinos, 2014). Although it remains unclear if a similar “closed-loop” exists in the cingulocerebellar circuit, we hope to elucidate these connections.

The cerebellar afferent pathway in the cingulocerebellar circuit involves direct anterior cingulate projections to the PN (Vilensky and Van Hoesen, 1981; Glickstein et al., 1985; Legg et al., 1989; Dembrow et al., 2010). Specifically, Vilensky et al. report that the rostral cingulate projects to the medial PN while the caudal cingulate regions project more laterally (Vilensky and Van Hoesen, 1981). These corticopontine fibers form the middle cerebellar peduncle and project to the deep cerebellar nuclei and cerebellar cortex.

There is evidence for two efferent pathways through which deep cerebellar nuclei communicate with the anterior cingulate. The first efferent pathway involves a disynaptic connection between the deep cerebellar nuclei and contralateral thalamus (Magnotta et al., 2008; Strick et al., 2009). Medial thalamic nuclei have been shown to project to the anterior cingulate cortex (Hsu and Shyu, 1997; Parnaudeau et al., 2013) although there is evidence for projections from all thalamic nuclei depending on the precise location of the anterior cingulate/prelimbic area in question (Condé et al., 1990). Therefore, it is likely that cerebellar projections to the thalamus are capable of transmitting information to broad regions of the anterior cingulate cortex. These connections are highly topographic and the labeled neurons are highly dependent on the precise location of the tracer injection.

The second efferent pathway includes deep cerebellar nuclei (fastigial) projections to the VTA (Snider et al., 1976). The VTA is known to supply dopaminergic input to the anterior cingulate and is able to affect neuronal activity when pharmacologically manipulated (Williams and Goldman-Rakic, 1998). Studies have corroborated this efferent pathway by showing that electrically stimulating cerebellar dentate nuclei (DN; Mittleman et al., 2008) and fastigial nuclei (FN; Watson et al., 2009) influences medial prefrontal dopamine signaling, which is impaired in schizophrenia (Hadley et al., 2014). Conversely, electrically stimulating the medial prefrontal cortex elicited neuronal firing in cerebellar lobule VII which projects back to the deep nuclei (Watson et al., 2009). A similar pathway and mechanistic approach to target the dento-VTA-PFC tract has been suggested by Rogers et al. in autism (Rogers et al., 2013) (see section on applications for neuropsychiatric illnesses). One challenge of electrical stimulation in mapping these circuits is that the influence of fibers of passage and indirect stimulation of neighboring nuclei confounds the results. Future studies using optogenetics may achieve greater specificity.

**Figure 3:**
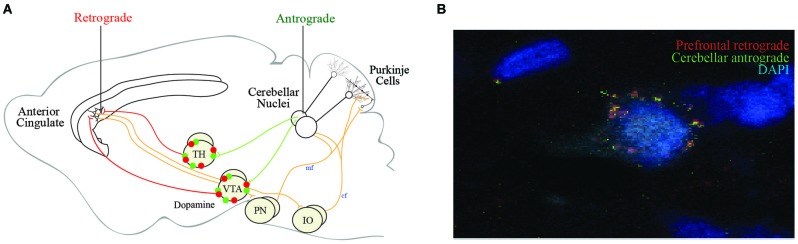
**Proposed efferent cingulocerellar circuitry**. **(A)** Schematic representation of the efferent cerebellar projections enabling cerebellar access to the medial prefrontal cortex/anterior cingulate via the ventrolateral thalamus and VTA. Two efferent pathways are thought to connect the cerebellum and prefrontal cortex (1) Cerebellar projections originating from dentate (DN) or fastigial nuclei (FN) to the contralateral thalamus and anterior cingulate cortex; and (2) Cerebellar projections originating from DN or FN to the contralateral ventral tegmental area (VTA) which send dopaminergic projections to the anterior cingulate cortex. The afferent pathways from the anterior cingulate back to the cerebellum via the pontine nuclei (PN) and inferior olive (IO). **(B)** Our tract tracing data following anterograde tracer (green) in the right dentate nuclei and retrograde tracer (red) in the contralateral (left) medial prefrontal cortex revealed tracer colocalization of both red and green beads on a single contralateral ventrolateral thalamic (VLTh) neuron **(B)**.

Although these studies show connections exist, the precise projections remain elusive. To illuminate the cingulocerebellar circuit, we infused retrograde tracer (RetroBeads, Lumaflour) in the left anterior cingulate/medial frontal cortex and anterograde tracer (Phaseolus vulgaris leucoagglutinin, Life Technologies) in the right cerebellar dentate deep nuclei of Long Evans rats and found these structures to be disynaptically connected via two distinct routes as previously reported. Single synapse red prefrontal beads and green cerebellar tracer colocalized on left ventrolateral thalamic nuclei and on VTA neurons nuclei (Figures 3A,B). Convergence of information in these networks provides two avenues through which the cerebellum may influence neurons in the anterior cingulate cortex. However, the cerebellum may influence cortical function indirectly through other thalamic inputs. More analyses are needed to identify or rule out colocalization in other thalamic nuclei.

This anatomical connection opens up an avenue for the cerebellum to modulate aberrant prefrontal networks in schizophrenia. We are currently analyzing longitudinal DTI and tractography data from patients at intake and throughout the disease course, which will allow us to choose regions of interest in the anterior cingulate and the deep cerebellar nuclei to more precisely document abnormalities in the cingulocerebellar circuit. It is important to note that as deep cerebellar nuclei are the sole output of the cerebellum, abnormalities in any area of the cerebellum have the potential to influence frontal function through the cinglocerebellar circuit.

#### A testable hypothesis: cerebellar stimulation restores prefrontal function and rescues cognition in schizophrenia

It is through the convergence of cerebellar deep nuclei and anterior cingulate projections on thalamic neurons that we propose the cerebellum can be harnessed to rescue aberrant prefrontal circuits in schizophrenia. The implications of this efferent, disynaptic pathway are numerous. Cerebellar stimulation may have the ability to restore prefrontal neuronal activity and firing patterns, allowing relief from some of the cognitive symptoms of schizophrenia.

Several studies provide support for the efficacy of cerebellar stimulation in neuropsychiatry (Grimaldi et al., 2014). A classic study electrically stimulated the cerebellum and reported improved cognition based on increased alertness, improvement in thinking, and fluency of speech in addition to many enriched emotional characteristics in patients with epilepsy (Cooper et al., 1976). Recently, cerebellar vermal TBS has been effective at relieving some cognitive symptoms in treatment-resistant schizophrenia patients (Demirtas-Tatlidede et al., 2010). In addition, Schutter et al. showed that cerebellar vermal TBS produced downstream changes in neuronal activity in the frontal cortex (Schutter et al., 2003). The exact cerebellar circuitry needs to be explicitly mapped prior to pursuing translational research.

How the cerebellum normally influences the prefrontal cortex and anterior cingulate is an important question that can be investigated using neuronal ensemble recordings of these areas in animals exhibiting phenotypes of schizophrenia. The cingulocerebellar circuit is likely essential for a variety of cognitive tasks as is documented by the previously described neuroimaging results. Correlating neuronal recordings will provide clarity as to how the cerebellum and prefrontal cortex work in synchrony during cognitive tasks such as learning, timing, and attention. Task-specific modulation will indicate if these areas are necessary.

**Figure 4:**
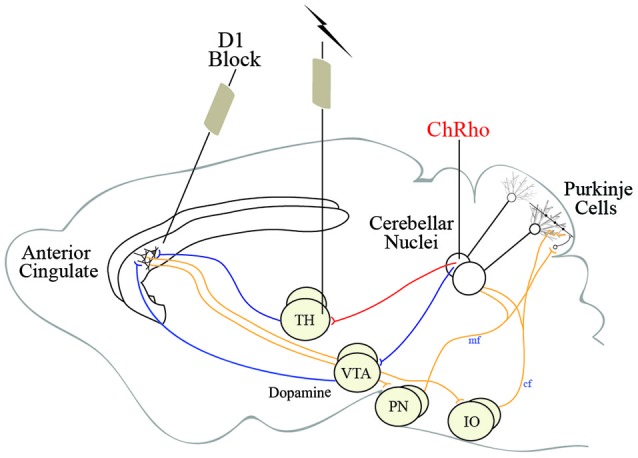
**Schematic representation of the cingulocerebellar pathways allowing the cerebellum access to the prefrontal cortex**. We propose using optogenetic stimulation of cerebellar projection neurons in the thalamus to recover activity in aberrant prefrontal neuronal ensembles in schizophrenia. Channelrhodopsin, a light-activated channel, can be infused into cerebellar neurons rendering cerebellar projections photoexcitable. Stimulating thalamic (or VTA) optical fibers can selectively stimulate the cerebellar neuronal projections to the anterior cingulate while not affecting other cerebellar neuronal populations. This optogenetic paradigm can be used in animals exhibiting phenotypes of schizophrenia and other neuropsychiatric illnesses in combination with elementary cognitive tasks impaired in schizophrenia to recover cognitive function and probe the cingluocerebellar circuit.

Several groups have made progress deciphering the role of nodes in the cingulocerebellar circuit using electrical stimulation (Mittleman et al., 2008; Watson et al., 2009; Rogers et al., 2011, 2013). However, results from these studies are limited due to the unwanted spread of electrical stimulation. To circumvent this issue, we propose using optogenetic stimulation of select, isolated pools of neurons in the cingulocerebellar circuit. Optogenetic stimulation of Purkinje cells has been shown to be an effective way to modulate cerebellar output (Tsubota et al., 2011). Using our tract-tracing results to target specific deep nuclear projections in the cingluocerebellar circuit, channelrhodopsin, a light-activated channel, can be infused into cerebellar neurons. Once expressed, these proteins render their projections photoexcitable. Optical fibers can be placed in the thalamus to selectively stimulate the cerebellar neuronal projections to the anterior cingulate while not affecting other cerebellar neuronal populations. Optogenetically stimulating cerebellar dentate projections in the thalamus could influence the prefrontal cortex (Figure 4). This optogenetic paradigm could be combined with neuronal ensemble recordings to probe the dynamic relationship between the prefrontal cortex and cerebellum while analyzing task-dependent modulation. Based on evidence from cerebellar stimulation studies, cerebellar optogenetic stimulation has the potential to enhance prefrontal neuronal modulation and show correlates of behavioral improvement.

Another way to investigate the cingulocerebellar circuit is to induce abnormalities similar to those reported during schizophrenia and attempt to rescue impairments using cerebellar stimulation. Targeting neurotransmitter cascades such as dopamine (Abi-Dargham et al., 2002; Goldman-Rakic et al., 2004), glutamate (Olney and Farber, 1995; Chaki and Hikichi, 2011; Moghaddam and Javitt, 2012; Anticevic et al., 2013), and GABA in prefrontal pyramidal neurons, it is possible to mimic schizophrenia phenotypes by direct pharmacological microinjections (Jones et al., 2011). These manipulations may induce changes in neuronal activity that resemble abnormalities in schizophrenia and can be correlated with behavioral abnormalities. For example, frontal dopamine has been implicated in schizophrenia (Abi-Dargham et al., 2002; Goldman-Rakic et al., 2004; Stahl and Buckley, 2007). The level of frontal dopamine determines the type and severity of associated symptoms (Goldman-Rakic et al., 2004; Kuepper et al., 2012). Excess dopamine contributes to positive symptoms such as hallucinations and delusions, while depleted dopamine is responsible for negative symptoms such as anhedonia, avolition, and cognitive deficits such as impaired timing (Andreasen and Olsen, 1982; Seeman, 1987; Kuepper et al., 2012).

We, and others, have shown that temporal processing depends on the cerebellum for short, sub-second intervals (Ivry and Spencer, 2004; Buhusi and Meck, 2005; Bracha et al., 2009; Parker et al., 2009) while D1 dopamine signaling in the anterior cingulate of the prefrontal cortex is essential for longer intervals (Deutch, 1993; Narayanan et al., 2012; Parker et al., 2013a,c). In schizophrenia, the prefrontal cortex shows abnormal D1 dopamine (Weinberger et al., 1986; Okubo et al., 1997; Goldman-Rakic et al., 2004) and patients inaccurately estimate a discrete interval of time (Elvevåg et al., 2003; Bonnot et al., 2011). In animals, it is possible to model these concepts by locally infusing pharmacological agents into the frontal cortex to disrupt prefrontal D1 dopamine signaling and mimic impaired timing (Narayanan et al., 2012). If the cingulocerebellar circuit is necessary for temporal processing and is sensitive to levels of prefrontal dopamine, pharmacologically manipulating prefrontal dopamine will disrupt neuronal activity and impair timing. Mittleman et al. report efflux of dopamine in the prelimibic cortex (anterior cingulate) following electrical stimulation of the dentate nuclei (Mittleman et al., 2008). Therefore, optogenetically stimulating cerebellar nuclei has the potential to rescue aberrant contralateral prefrontal neuronal ensembles, recovering elementary cognitive tasks (Boyden et al., 2005; Narayanan et al., 2012).

An example of a task that has been used to probe the role of the cingulocerebellar circuit is eyeblink conditioning as it is abnormal in schizophrenia (Brown et al., 2005; Bolbecker et al., 2009; Forsyth et al., 2012; Parker et al., 2013b). Specifically, trace eyeblink conditioning, where a conditioning stimulus and an unconditioned stimulus are separated by a trace interval, requires integration of the cerebellum and frontal cortex (Weiss and Disterhoft, 1996). Siegel et al. have explored this afferent pathway and the influence of the dorsal anterior cingulate in layers V and VII (secondary motor cortices) in rabbits (Siegel et al., 2012). They found direct projections to the ipsilateral rostral PN via the corticospinal pyramidal tract. It has also been reported that prefrontal neurons are consistently active in the trace interstimulus interval, likely encoding the elapsed time between the stimulus and providing the cerebellum with the timing information necessary to accurately execute the eyeblink response (Siegel et al., 2012; Chen et al., 2014). It is possible to pharmacologically mimic psychiatric abnormalities by inactivating various areas of the prefrontal cortex to produce disruptions in trace eyeblink conditioning. Following expression of channelrhodopsin infusions in deep cerebellar nuclei, optogenetic stimulation of cerebellar projections to the thalamus have the potential to recover prefrontal neuronal activity and rescue eyeblink conditioning.

Optogenetic stimulation of the cingulocerebellar pathway can also be explored in genetically modified animals exhibiting schizophrenia phenotypes. Currently, several animals exist that exhibit some phenotypes of schizophrenia such as G72/G30, Df(16)A KO model, and Disrupted in Schizophrenia 1 (DISC1; Shevelkin et al., 2014). Each of these animals has cerebellar abnormalities similar to those consistently detected in schizophrenia making them ideal models to study the cingulocerebellar circuit (Shevelkin et al., 2014).

There are many additional aspects of this circuit that can be explored. Although we have focused on a very specific circuit involving the anterior cingulate and cerebellum, it is possible that a more widespread activity is sufficient for normal functioning. There are known connections throughout frontal lobes and normalized neuronal firing throughout the brain may prove essential rather than the firing patterns of one particular structure. This theory is supported by the more diffuse type of stimulation used by transcranial magnetic stimulation (Grimaldi et al., 2014) and thalamic stimulation (Klein et al., 2013).

### Cerebellar stimulation in parkinson’s disease and autism

Although schizophrenia is not considered to be a motor disease, many indicators of motor dysfunction are present, suggesting that the basic abnormality in the disorder could be a brain system that mediates both motor and cognitive functions. Kraepelin described a variety of motor abnormalities in schizophrenia in his classic textbook (1919). Slowed reaction time is one of the oldest and most robust tests showing differences between schizophrenic patients and normal controls. More recent studies of premorbid indicators and neurological soft signs also implicate the motor system (Walker and Shaye, 1982; Gupta et al., 1995; Flashman et al., 1996; Mouchet-Mages et al., 2011). These mild indicators of poor coordination provide a clue that the underlying mechanism may reflect an abnormality in a basic process that regulates the synchrony of both thought and motor activity. Therefore, schizophrenia may be conceptualized as a disease that is characterized by poor coordination, or dysmetria, in all domains of functioning, including movement and cognition. In addition to its role in cognition, the cerebellum has traditionally been associated with movement and gait. Therefore, the circuit between the prefrontal cortex and cerebellum may facilitate the smooth execution, or synchrony of both motor and cognitive function. As we have proposed optogenetic stimulation of cerebellar projections for the treatment of the cognitive symptoms of schizophrenia, the motor impairments may actually benefit as well.

Parkinson’s disease (PD) also causes impaired gait and cognition and involves abnormalities in the cerebellum, prefrontal cortex, and the basal ganglia. Cerebellar circuitry involves efferent connections with the basal ganglia which project back to the cerebellum via the PN (similar to the anterior cingulate projections) (Bostan and Strick, 2010; Bostan et al., 2010). However, similar to schizophrenia, the role of the cerebellum in PD is unknown. Recently, Wu et al. published a thorough review of the role of the cerebellum in PD (Wu and Hallett, 2013). They speculated that the cerebellum plays either a compensatory role or a pathological role (Wu and Hallett, 2013). Using optogenetic stimulation of cerebellar projections in the striatum, it may be possible to modulate cerebellar activity and repair movement, gait, and cognition in PD patients. How cerebellar circuitry interacts with the anterior cingulate via the dopaminergic VTA projections should be pursued, as cerebellar stimulation could represent novel therapeutic opportunities for both the cognitive and motor impairments in PD.

Courchesne et al. have documented cerebellar abnormalities in autism. In addition to decrease in cerebellar vermal volume, there appears to be aberrancies in cerebellar-prefrontal interactions (Carper and Courchesne, 2000; Pierce and Courchesne, 2001; Courchesne et al., 2011). Recently, Purkinje cell abnormalities have been linked to behavioral deficits similar to those exhibited in autism (Sudarov, 2013). The middle cerebellar peduncle has also shown structural impairments in autism and as the output pathway for cerebellar projections, this altered microstructure could have dire consequences for cerebellar-frontal lobe communications (Sivaswamy et al., 2010; Hanaie et al., 2013). More research needs to be done to understand the behavioral and cognitive symptoms in autism which depend on the cingulocerebellar pathway (Heck and Howell, 2013). Using optogenetics, this circuit should be parsed out and pursued to repair brain circuitry in autism. There are currently several animal models that exhibit phenotypes of autism, which are ripe for investigation.

## Conclusions

The ideas described here have the potential to map the influence of cerebellar circuitry on the frontal cortex and lead to new insights about cingulocerebellar interactions. Through the cingulocerebellar circuit, cerebellar stimulation may recover aberrancies in the anterior cingulate cortex, rescuing cognition in schizophrenia. To our knowledge, this idea has not been proposed before, and we hope systematically applying the techniques described here to the many cognitive tasks impaired in schizophrenia such as attention and working memory, will encourage the development of new, targeted treatments for treatment-resistant patients.

## Conflict of interest statement

The authors declare that the research was conducted in the absence of any commercial or financial relationships that could be construed as a potential conflict of interest.

## References

[B1] Abi-Dargham A. Mawlawi O. Lombardo I. Gil R. Martinez D. Huang Y. (2002). Prefrontal dopamine D1 receptors and working memory in schizophrenia. J. Neurosci. 22, 3708–3719 11978847 10.1523/JNEUROSCI.22-09-03708.2002 PMC6758376

[B2] Adams R. David A. S. (2007). Patterns of anterior cingulate activation in schizophrenia: a selective review. Neuropsychiatr. Dis. Treat. 3, 87–101 10.2147/nedt.2007.3.1.87 19300540 PMC2654525

[B3] Akshoomoff N. A. Courchesne E. (1992). A new role for the cerebellum in cognitive operations. Behav. Neurosci. 106, 731–738 10.1037/0735-7044.106.5.731 1445653

[B4] Alphs L. (2006). An industry perspective on the NIMH consensus statement on negative symptoms. Schizophr. Bull. 32, 225–230 10.1093/schbul/sbj056 16469940 PMC2632231

[B5] Andreasen N. C. Arndt S. Swayze V. 2nd Cizadlo T. Flaum M. O’Leary D. (1994). Thalamic abnormalities in schizophrenia visualized through magnetic resonance image averaging. Science 266, 294–298 10.1126/science.7939669 7939669

[B6] Andreasen N. C. O’Leary D. S. Arndt S. Cizadlo T. Rezai K. Watkins G. L. (1995a). I. PET studies of memory: novel and practiced free recall of complex narratives. Neuroimage 2, 284–295 10.1006/nimg.1995.1036 9343613

[B7] Andreasen N. C. O’Leary D. S. Cizadlo T. Arndt S. Rezai K. Watkins G. L. (1995b). II. PET studies of memory: novel versus practiced free recall of word lists. Neuroimage 2, 296–305 10.1006/nimg.1995.1037 9343614

[B8] Andreasen N. C. O’Leary D. S. Flaum M. Nopoulos P. Watkins G. L. Boles Ponto L. L. (1997). Hypofrontality in schizophrenia: distributed dysfunctional circuits in neuroleptic-naïve patients. Lancet 349, 1730–1734 10.1016/s0140-6736(96)08258-x 9193383

[B9] Andreasen N. C. O’Leary D. S. Paradiso S. Cizadlo T. Arndt S. Watkins G. L. (1999). The cerebellum plays a role in conscious episodic memory retrieval. Hum. Brain Mapp. 8, 226–234 10.1002/(sici)1097-0193(1999)8:4<226::aid-hbm6>3.0.co;2-4 10619416 PMC6873320

[B10] Andreasen N. C. Olsen S. (1982). Negative v positive schizophrenia: definition and validation. Arch. Gen. Psychiatry 39, 789–794 10.1001/archpsyc.1982.04290070025006 7165478

[B11] Andreasen N. C. Paradiso S. O’Leary D. S. (1998). “Cognitive Dysmetria” as an integrative theory of schizophrenia. Schizophr. Bull. 24, 203–218 10.1093/oxfordjournals.schbul.a033321 9613621

[B12] Andreasen N. C. Pierson R. (2008). The role of the cerebellum in schizophrenia. Biol. Psychiatry 64, 81–88 10.1016/j.biopsych.2008.01.003 18395701 PMC3175494

[B13] Anticevic A. Cole M. W. Repovs G. Savic A. Driesen N. R. Yang G. (2013). Connectivity, pharmacology and computation: toward a mechanistic understanding of neural system dysfunction in schizophrenia. Front. Psychiatry 4:169 10.3389/fpsyt.2013.00169 24399974 PMC3871997

[B14] Artiges E. Salamé P. Recasens C. Poline J. B. Attar-Levy D. De La Raillère A. (2000). Working memory control in patients with schizophrenia: a PET study during a random number generation task. Am. J. Psychiatry 157, 1517–1519 10.1176/appi.ajp.157.9.1517 10964875

[B15] Bijsterbosch J. D. Lee K.-H. Hunter M. D. Tsoi D. T. Lankappa S. Wilkinson I. D. (2011). The role of the cerebellum in sub- and supraliminal error correction during sensorimotor synchronization: evidence from fMRI and TMS. J. Cogn. Neurosci. 23, 1100–1112 10.1162/jocn.2010.21506 20465354

[B16] Boehringer A. Macher K. Dukart J. Villringer A. Pleger B. (2013). Cerebellar transcranial direct current stimulation modulates verbal working memory. Brain Stimul. 6, 649–653 10.1016/j.brs.2012.10.001 23122917

[B17] Bolbecker A. R. Mehta C. S. Edwards C. R. Steinmetz J. E. O’Donnell B. F. Hetrick W. P. (2009). Eye-blink conditioning deficits indicate temporal processing abnormalities in schizophrenia. Schizophr. Res. 111, 182–191 10.1016/j.schres.2009.03.016 19351577 PMC2702657

[B18] Bonnot O. de Montalembert M. Kermarrec S. Botbol M. Walter M. Coulon N. (2011). Are impairments of time perception in schizophrenia a neglected phenomenon? J. Physiol. Paris 105, 164–169 10.1016/j.jphysparis.2011.07.006 21803155

[B19] Bostan A. C. Dum R. P. Strick P. L. (2010). The basal ganglia communicate with the cerebellum. Proc. Natl. Acad. Sci. U S A 107, 8452–8456 10.1073/pnas.1000496107 20404184 PMC2889518

[B20] Bostan A. C. Dum R. P. Strick P. L. (2013). Cerebellar networks with the cerebral cortex and basal ganglia. Trends Cogn. Sci. 17, 241–254 10.1016/j.tics.2013.03.003 23579055 PMC3645327

[B21] Bostan A. C. Strick P. L. (2010). The cerebellum and basal ganglia are interconnected. Neuropsychol. Rev. 20, 261–270 10.1007/s11065-010-9143-9 20811947 PMC3325093

[B22] Boyden E. S. Zhang F. Bamberg E. Nagel G. Deisseroth K. (2005). Millisecond-timescale, genetically targeted optical control of neural activity. Nat. Neurosci. 8, 1263–1268 10.1038/nn1525 16116447

[B23] Bracha V. Zbarska S. Parker K. Carrel A. Zenitsky G. Bloedel J. R. (2009). The cerebellum and eye-blink conditioning: learning versus network performance hypotheses. Neuroscience 162, 787–796 10.1016/j.neuroscience.2008.12.042 19162131 PMC2822538

[B24] Brown S. M. Kieffaber P. D. Carroll C. A. Vohs J. L. Tracy J. A. Shekhar A. (2005). Eyeblink conditioning deficits indicate timing and cerebellar abnormalities in schizophrenia. Brain Cogn. 58, 94–108 10.1016/j.bandc.2004.09.011 15878730

[B25] Buhusi C. V. Meck W. H. (2005). What makes us tick? Functional and neural mechanisms of interval timing. Nat. Rev. Neurosci. 6, 755–765 10.1038/nrn1764 16163383

[B26] Buschman T. J. Miller E. K. (2007). Top-down versus bottom-up control of attention in the prefrontal and posterior parietal cortices. Science 315, 1860–1862 10.1126/science.1138071 17395832

[B27] Carper R. A. Courchesne E. (2000). Inverse correlation between frontal lobe and cerebellum sizes in children with autism. Brain 123(Pt. 4), 836–844 10.1093/brain/123.4.836 10734014

[B28] Carroll C. A. O’Donnell B. F. Shekhar A. Hetrick W. P. (2009). Timing dysfunctions in schizophrenia as measured by a repetitive finger tapping task. Brain Cogn. 71, 345–353 10.1016/j.bandc.2009.06.009 19664870 PMC2783288

[B29] Carter C. S. Braver T. S. Barch D. M. Botvinick M. M. Noll D. Cohen J. D. (1998). Anterior cingulate cortex, error detection and the online monitoring of performance. Science 280, 747–749 10.1126/science.280.5364.747 9563953

[B30] Carter C. S. MacDonald A. W. 3rd Ross L. L. Stenger V. A. (2001). Anterior cingulate cortex activity and impaired self-monitoring of performance in patients with schizophrenia: an event-related fMRI study. Am. J. Psychiatry 158, 1423–1428 10.1176/appi.ajp.158.9.1423 11532726

[B31] Cavanagh J. F. Cohen M. X. Allen J. J. B. (2009). Prelude to and resolution of an error: EEG phase synchrony reveals cognitive control dynamics during action monitoring. J. Neurosci. 29, 98–105 10.1523/jneurosci.4137-08.2009 19129388 PMC2742325

[B32] Chaki S. Hikichi H. (2011). Targeting of metabotropic glutamate receptors for the treatment of schizophrenia. Curr. Pharm. Des. 17, 94–102 10.2174/138161211795049570 21355835

[B33] Chen H. Yang L. Xu Y. Wu G. Yao J. Zhang J. (2014). Prefrontal control of cerebellum-dependent associative motor learning. Cerebellum 13, 64–78 10.1007/s12311-013-0517-4 24013852

[B34] Condé F. Audinat E. Maire-Lepoivre E. Crépel F. (1990). Afferent connections of the medial frontal cortex of the rat. A study using retrograde transport of fluorescent dyes. I. Thalamic afferents. Brain Res. Bull. 24, 341–354 10.1016/0361-9230(90)90088-h 2337814

[B35] Cooper I. S. Amin I. Riklan M. Waltz J. M. Poon T. P. (1976). Chronic cerebellar stimulation in epilepsy. Clinical and anatomical studies. Arch. Neurol. 33, 559–570 10.1001/archneur.1976.00500080037006 821458

[B36] Courchesne E. Mouton P. R. Calhoun M. E. Semendeferi K. Ahrens-Barbeau C. Hallet M. J. (2011). Neuron number and size in prefrontal cortex of children with autism. JAMA 306, 2001–2010 10.1001/jama.2011.1638 22068992

[B37] Courchesne E. Townsend J. Akshoomoff N. A. Saitoh O. Yeung-Courchesne R. Lincoln A. J. (1994). Impairment in shifting attention in autistic and cerebellar patients. Behav. Neurosci. 108, 848–865 10.1037/0735-7044.108.5.848 7826509

[B38] Crespo-Facorro B. Paradiso S. Andreasen N. C. O’Leary D. S. Watkins G. L. Boles Ponto L. L. (1999). Recalling word lists reveals “cognitive dysmetria” in schizophrenia: a positron emission tomography study. Am. J. Psychiatry 156, 386–392 10080553 10.1176/ajp.156.3.386

[B39] Crespo-Facorro B. Wiser A. K. Andreasen N. C. O’Leary D. S. Watkins G. L. Boles Ponto L. L. (2001). Neural basis of novel and well-learned recognition memory in schizophrenia: a positron emission tomography study. Hum. Brain Mapp. 12, 219–231 10.1002/1097-0193(200104)12:4<219::aid-hbm1017>3.0.co;2-l 11241873 PMC6871838

[B40] Dembrow N. C. Chitwood R. A. Johnston D. (2010). Projection-specific neuromodulation of medial prefrontal cortex neurons. J. Neurosci. 30, 16922–16937 10.1523/jneurosci.3644-10.2010 21159963 PMC3075873

[B41] Demirtas-Tatlidede A. Freitas C. Cromer J. R. Safar L. Ongur D. Stone W. S. (2010). Safety and proof of principle study of cerebellar vermal theta burst stimulation in refractory schizophrenia. Schizophr. Res. 124, 91–100 10.1016/j.schres.2010.08.015 20817483 PMC3268232

[B42] Demirtas-Tatlidede A. Vahabzadeh-Hagh A. M. Pascual-Leone A. (2013). Can noninvasive brain stimulation enhance cognition in neuropsychiatric disorders? Neuropharmacology 64, 566–578 10.1016/j.neuropharm.2012.06.020 22749945 PMC3725288

[B43] Deutch A. Y. (1993). Prefrontal cortical dopamine systems and the elaboration of functional corticostriatal circuits: implications for schizophrenia and Parkinson’s disease. J. Neural Transm. Gen. Sect. 91, 197–221 10.1007/bf01245232 8099797

[B44] Devinsky O. Morrell M. J. Vogt B. A. (1995). Contributions of anterior cingulate cortex to behaviour. Brain 118(Pt. 1), 279–306 10.1093/brain/118.1.279 7895011

[B45] Elvevåg B. McCormack T. Gilbert A. Brown G. D. A. Weinberger D. R. Goldberg T. E. (2003). Duration judgements in patients with schizophrenia. Psychol. Med. 33, 1249–1261 10.1017/s0033291703008122 14580079

[B46] Fierro B. Palermo A. Puma A. Francolini M. Panetta M. L. Daniele O. (2007). Role of the cerebellum in time perception: a TMS study in normal subjects. J. Neurol. Sci. 263, 107–112 10.1016/j.jns.2007.06.033 17655867

[B47] Fiez J. A. Petersen S. E. Cheney M. K. Raichle M. E. (1992). Impaired non-motor learning and error detection associated with cerebellar damage. A single case study. Brain 115(Pt. 1), 155–178 10.1093/brain/115.1.155 1559151

[B48] Flashman L. A. Flaum M. Gupta S. Andreasen N. C. (1996). Soft signs and neuropsychological performance in schizophrenia. Am. J. Psychiatry 153, 526–532 8599401 10.1176/ajp.153.4.526

[B49] Fornito A. Yücel M. Dean B. Wood S. J. Pantelis C. (2009). Anatomical abnormalities of the anterior cingulate cortex in schizophrenia: bridging the gap between neuroimaging and neuropathology. Schizophr. Bull. 35, 973–993 10.1093/schbul/sbn025 18436528 PMC2728810

[B50] Forsyth J. K. Bolbecker A. R. Mehta C. S. Klaunig M. J. Steinmetz J. E. O’Donnell B. F. (2012). Cerebellar-dependent eyeblink conditioning deficits in schizophrenia spectrum disorders. Schizophr. Bull. 38, 751–759 10.1093/schbul/sbQ128 21148238 PMC3406528

[B51] Gasquoine P. G. (2013). Localization of function in anterior cingulate cortex: from psychosurgery to functional neuroimaging. Neurosci. Biobehav. Rev. 37, 340–348 10.1016/j.neubiorev.2013.01.002 23313645

[B52] Glahn D. C. Laird A. R. Ellison-Wright I. Thelen S. M. Robinson J. L. Lancaster J. L. (2008). Meta-analysis of gray matter anomalies in schizophrenia: application of anatomic likelihood estimation and network analysis. Biol. Psychiatry 64, 774–781 10.1016/j.biopsych.2008.03.031 18486104 PMC5441233

[B53] Glickstein M. May J. G. 3rd Mercier B. E. (1985). Corticopontine projection in the macaque: the distribution of labelled cortical cells after large injections of horseradish peroxidase in the pontine nuclei. J. Comp. Neurol. 235, 343–359 10.1002/cne.902350306 3998215

[B54] Goldman-Rakic P. S. Castner S. A. Svensson T. H. Siever L. J. Williams G. V. (2004). Targeting the dopamine D1 receptor in schizophrenia: insights for cognitive dysfunction. Psychopharmacology (Berl) 174, 3–16 10.1007/s00213-004-1793-y 15118803

[B55] Gottwald B. Wilde B. Mihajlovic Z. Mehdorn H. M. (2004). Evidence for distinct cognitive deficits after focal cerebellar lesions. J. Neurol. Neurosurg. Psychiatry 75, 1524–1531 10.1136/jnnp.2003.018093 15489381 PMC1738803

[B56] Grafman J. Litvan I. Massaquoi S. Stewart M. Sirigu A. Hallett M. (1992). Cognitive planning deficit in patients with cerebellar atrophy. Neurology 42, 1493–1496 10.1212/wnl.42.8.1493 1641142

[B57] Grimaldi G. Argyropoulos G. P. Boehringer A. Celnik P. Edwards M. J. Ferrucci R. (2014). Non-invasive cerebellar stimulation-a consensus paper. Cerebellum 13, 121–138 10.1007/s12311-013-0514-7 23943521

[B58] Grimaldi G. Manto M. (2012). Topography of cerebellar deficits in humans. Cerebellum 11, 336–351 10.1007/s12311-011-0247-4 21240580

[B59] Groiss S. J. Ugawa Y. (2013). “Chapter 51—Cerebellum,” in Handbook of Clinical Neurology. Brain Stimulation, eds Lozano A. M. Hallett M. (Elsevier), 643–653 Available online at: http://www.sciencedirect.com/science/article/pii/B9780444534972000516 Accessed on February 21 2014. 10.1016/B978-0-444-53497-2.00051-6 24112930

[B60] Grube M. Lee K.-H. Griffiths T. D. Barker A. T. Woodruff P. W. (2010). Transcranial magnetic theta-burst stimulation of the human cerebellum distinguishes absolute, duration-based from relative, beat-based perception of subsecond time intervals. Front. Psychol. 1:171 10.3389/fpsyg.2010.00171 21833234 PMC3153783

[B61] Gupta S. Andreasen N. C. Arndt S. Flaum M. Schultz S. K. Hubbard W. C. (1995). Neurological soft signs in neuroleptic-naive and neuroleptic-treated schizophrenic patients and in normal comparison subjects. Am. J. Psychiatry 152, 191–196 7840351 10.1176/ajp.152.2.191

[B62] Hadley J. A. Nenert R. Kraguljac N. V. Bolding M. S. White D. M. Skidmore F. M. (2014). Ventral tegmental area/midbrain functional connectivity and response to antipsychotic medication in schizophrenia. Neuropsychopharmacology 39, 1020–1030 10.1038/npp.2013.305 24165885 PMC3924537

[B63] Hanaie R. Mohri I. Kagitani-Shimono K. Tachibana M. Azuma J. Matsuzaki J. (2013). Altered microstructural connectivity of the superior cerebellar peduncle is related to motor dysfunction in children with autistic spectrum disorders. Cerebellum 12, 645–656 10.1007/s12311-013-0475-x 23564050

[B64] Heck D. H. Howell J. W. (2013). Prefrontal cortical-cerebellar interaction deficits in autism spectrum disorders. *Autism* S4:001. 10.4172/2165-7890.S4-001

[B65] Honey G. D. Pomarol-Clotet E. Corlett P. R. Honey R. A. E. McKenna P. J. Bullmore E. T. (2005). Functional dysconnectivity in schizophrenia associated with attentional modulation of motor function. Brain 128, 2597–2611 10.1093/brain/awh632 16183659 PMC3838931

[B66] Hsu M. M. Shyu B. C. (1997). Electrophysiological study of the connection between medial thalamus and anterior cingulate cortex in the rat. Neuroreport 8, 2701–2707 10.1097/00001756-199708180-00013 9295104

[B67] Ivry R. B. Spencer R. M. (2004). Evaluating the role of the cerebellum in temporal processing: beware of the null hypothesis. Brain 127, E13–E14 10.1093/brain/awh227 15277307

[B68] Jones C. Watson D. Fone K. (2011). Animal models of schizophrenia. Br. J. Pharmacol. 164, 1162–1194 10.1111/j.1476-5381.2011.01386.x 21449915 PMC3229756

[B69] Kamali A. Kramer L. A. Frye R. E. Butler I. J. Hasan K. M. (2010). Diffusion tensor tractography of the human brain cortico-ponto-cerebellar pathways: a quantitative preliminary study. J. Magn. Reson. Imaging 32, 809–817 10.1002/jmri.22330 20882611 PMC4492525

[B70] Klein J. Hadar R. Götz T. Männer A. Eberhardt C. Baldassarri J. (2013). Mapping brain regions in which deep brain stimulation affects schizophrenia-like behavior in two rat models of schizophrenia. Brain Stimul. 6, 490–499 10.1016/j.brs.2012.09.004 23085443

[B71] Koch G. Oliveri M. Torriero S. Salerno S. Lo Gerfo E. Caltagirone C. (2007). Repetitive TMS of cerebellum interferes with millisecond time processing. Exp. Brain Res. 179, 291–299 10.1007/s00221-006-0791-1 17146647

[B72] Koziol L. F. Budding D. Andreasen N. D’Arrigo S. Bulgheroni S. Imamizu H. (2013). Consensus paper: the cerebellum’s role in movement and cognition. Cerebellum 13, 151–177 10.1007/s12311-013-0511-x 23996631 PMC4089997

[B73] Kuepper R. Skinbjerg M. Abi-Dargham A. (2012). The dopamine dysfunction in schizophrenia revisited: new insights into topography and course. Handb. Exp. Pharmacol. 212, 1–26 10.1007/978-3-642-25761-2_1 23129326

[B74] Kühn S. Romanowski A. Schubert F. Gallinat J. (2012). Reduction of cerebellar grey matter in Crus I and II in schizophrenia. Brain Struct. Funct. 217, 523–529 10.1007/s00429-011-0365-2 22131119

[B75] Legg C. R. Mercier B. Glickstein M. (1989). Corticopontine projection in the rat: the distribution of labelled cortical cells after large injections of horseradish peroxidase in the pontine nuclei. J. Comp. Neurol. 286, 427–441 10.1002/cne.902860403 2778100

[B76] Leiner H. C. Leiner A. L. Dow R. S. (1994). The underestimated cerebellum. Hum. Brain Mapp. 2, 244–254 10.1002/hbm.460020406

[B77] Magnotta V. A. Adix M. L. Caprahan A. Lim K. Gollub R. Andreasen N. C. (2008). Investigating connectivity between the cerebellum and thalamus in schizophrenia using diffusion tensor tractography: a pilot study. Psychiatry Res. 163, 193–200 10.1016/j.pscychresns.2007.10.005 18656332 PMC3847814

[B78] McCormick L. Decker L. Nopoulos P. Ho B.-C. Andreasen N. (2005). Effects of atypical and typical neuroleptics on anterior cingulate volume in schizophrenia. Schizophr. Res. 80, 73–84 10.1016/j.schres.2005.06.022 16169191

[B79] Meyer-Lindenberg A. Poline J. B. Kohn P. D. Holt J. L. Egan M. F. Weinberger D. R. (2001). Evidence for abnormal cortical functional connectivity during working memory in schizophrenia. Am. J. Psychiatry 158, 1809–1817 10.1176/appi.ajp.158.11.1809 11691686

[B80] Middleton F. A. Strick P. L. (2001). Cerebellar projections to the prefrontal cortex of the primate. J. Neurosci. 21, 700–712 11160449 10.1523/JNEUROSCI.21-02-00700.2001 PMC6763818

[B81] Minzenberg M. J. Laird A. R. Thelen S. Carter C. S. Glahn D. C. (2009). Meta-analysis of 41 functional neuroimaging studies of executive function in schizophrenia. Arch. Gen. Psychiatry 66, 811–822 10.1001/archgenpsychiatry.2009.91 19652121 PMC2888482

[B82] Mitelman S. A. Newmark R. E. Torosjan Y. Chu K.-W. Brickman A. M. Haznedar M. M. (2006). White matter fractional anisotropy and outcome in schizophrenia. Schizophr. Res. 87, 138–159 10.1016/j.schres.2006.06.016 16854563

[B83] Mittleman G. Goldowitz D. Heck D. H. Blaha C. D. (2008). Cerebellar modulation of frontal cortex dopamine efflux in mice: relevance to autism and schizophrenia. Synapse 62, 544–550 10.1002/syn.20525 18435424 PMC3854870

[B84] Moghaddam B. Javitt D. (2012). From revolution to evolution: the glutamate hypothesis of schizophrenia and its implication for treatment. Neuropsychopharmacology 37, 4–15 10.1038/npp.2011.181 21956446 PMC3238069

[B85] Mouchet-Mages S. Rodrigo S. Cachia A. Mouaffak F. Olie J. P. Meder J. F. (2011). Correlations of cerebello-thalamo-prefrontal structure and neurological soft signs in patients with first-episode psychosis. Acta Psychiatr. Scand. 123, 451–458 10.1111/j.1600-0447.2010.01667.x 21219267

[B86] Narayanan N. S. Cavanagh J. F. Frank M. J. Laubach M. (2013). Common medial frontal mechanisms of adaptive control in humans and rodents. Nat. Neurosci. 16, 1888–1895 10.1038/nn.3549 24141310 PMC3840072

[B87] Narayanan N. S. Land B. B. Solder J. E. Deisseroth K. Dileone R. J. (2012). Prefrontal D1 dopamine signaling is required for temporal control. Proc. Natl. Acad. Sci. U S A 109, 20726–20731 10.1073/pnas.1211258109 23185016 PMC3528521

[B88] Narayanan N. S. Prabhakaran V. Bunge S. A. Christoff K. Fine E. M. Gabrieli J. D. E. (2005). The role of the prefrontal cortex in the maintenance of verbal working memory: an event-related FMRI analysis. Neuropsychology 19, 223–232 10.1037/0894-4105.19.2.223 15769206

[B89] Nopoulos P. C. Ceilley J. W. Gailis E. A. Andreasen N. C. (1999). An MRI study of cerebellar vermis morphology in patients with schizophrenia: evidence in support of the cognitive dysmetria concept. Biol. Psychiatry 46, 703–711 10.1016/s0006-3223(99)00093-1 10472423

[B91] Oh J. S. Kubicki M. Rosenberger G. Bouix S. Levitt J. J. McCarley R. W. (2009). Thalamo-frontal white matter alterations in chronic schizophrenia: a quantitative diffusion tractography study. Hum. Brain Mapp. 30, 3812–3825 10.1002/hbm.20809 19449328 PMC2767408

[B92] Okubo Y. Suhara T. Suzuki K. Kobayashi K. Inoue O. Terasaki O. (1997). Decreased prefrontal dopamine D1 receptors in schizophrenia revealed by PET. Nature 385, 634–636 10.1038/385634a0 9024661

[B93] Okugawa G. Nobuhara K. Sugimoto T. Kinoshita T. (2005). Diffusion tensor imaging study of the middle cerebellar peduncles in patients with schizophrenia. Cerebellum 4, 123–127 10.1080/14734220510007879 16035194

[B94] Okugawa G. Sedvall G. C. Agartz I. (2003). Smaller cerebellar vermis but not hemisphere volumes in patients with chronic schizophrenia. Am. J. Psychiatry 160, 1614–1617 10.1176/appi.ajp.160.9.1614 12944335

[B90] O’Leary D. S. Andreason N. C. Hurtig R. R. Hichwa R. D. Watkins G. L. Ponto L. L. (1996). A positron emission tomography study of binaurally and dichotically presented stimuli: effects of level of language and directed attention. Brain Lang. 53, 20–39 10.1006/brln.1996.0034 8722897

[B95] Oliveri M. Torriero S. Koch G. Salerno S. Petrosini L. Caltagirone C. (2007). The role of transcranial magnetic stimulation in the study of cerebellar cognitive function. Cerebellum 6, 95–101 10.1080/14734220701213421 17366271

[B96] Olney J. W. Farber N. B. (1995). Glutamate receptor dysfunction and schizophrenia. Arch. Gen. Psychiatry 52, 998–1007 10.1001/archpsyc.1995.03950240016004 7492260

[B97] Paradiso S. Crespo Facorro B. Andreasen N. C. O’Leary D. S. Watkins L. G. Boles Ponto L. (1997). Brain activity assessed with PET during recall of word lists and narratives. Neuroreport 8, 3091–3096 10.1097/00001756-199709290-00017 9331920

[B98] Parker K. L. Alberico S. L. Miller A. D. Narayanan N. S. (2013a). Prefrontal D1 dopamine signaling is necessary for temporal expectation during reaction time performance. Neuroscience 255, 246–254 10.1016/j.neuroscience.2013.09.057 24120554 PMC3856920

[B99] Parker K. L. Andreasen N. C. Liu D. Freeman J. H. O’Leary D. S. (2013b). Eyeblink conditioning in unmedicated schizophrenia patients: a positron emission tomography study. Psychiatry Res. 214, 402–409 10.1016/j.pscychresns.2013.07.006 24090512 PMC3980571

[B100] Parker K. L. Lamichhane D. Caetano M. S. Narayanan N. S. (2013c). Executive dysfunction in Parkinson’s disease and timing deficits. Front. Integr. Neurosci. 7:75 10.3389/fnint.2013.00075 24198770 PMC3813949

[B101] Parker K. L. Zbarska S. Carrel A. J. Bracha V. (2009). Blocking GABAA neurotransmission in the interposed nuclei: effects on conditioned and unconditioned eyeblinks. Brain Res. 1292, 25–37 10.1016/j.brainres.2009.07.053 19635470 PMC2823115

[B102] Parnaudeau S. O’Neill P.-K. Bolkan S. S. Ward R. D. Abbas A. I. Roth B. L. (2013). Inhibition of mediodorsal thalamus disrupts thalamofrontal connectivity and cognition. Neuron 77, 1151–1162 10.1016/j.neuron.2013.01.038 23522049 PMC3629822

[B103] Picton T. W. Stuss D. T. Alexander M. P. Shallice T. Binns M. A. Gillingham S. (2006). Effects of focal frontal lesions on response inhibition. Cereb. Cortex 17, 826–838 10.1093/cercor/bhk031 16699079

[B104] Pierce K. Courchesne E. (2001). Evidence for a cerebellar role in reduced exploration and stereotyped behavior in autism. Biol. Psychiatry 49, 655–664 10.1016/s0006-3223(00)01008-8 11313033

[B105] Pomarol-Clotet E. Canales-Rodríguez E. J. Salvador R. Sarró S. Gomar J. J. Vila F. (2010). Medial prefrontal cortex pathology in schizophrenia as revealed by convergent findings from multimodal imaging. Mol. Psychiatry 15, 823–830 10.1038/mp.2009.146 20065955 PMC2927029

[B106] Prabhakaran V. Rypma B. Narayanan N. S. Meier T. B. Austin B. P. Nair V. A. (2011). Capacity-speed relationships in prefrontal cortex. PLoS One 6:e27504 10.1371/journal.pone.0027504 22132105 PMC3223164

[B107] Ragland J. D. Laird A. R. Ranganath C. Blumenfeld R. S. Gonzales S. M. Glahn D. C. (2009). Prefrontal activation deficits during episodic memory in schizophrenia. Am. J. Psychiatry 166, 863–874 10.1176/appi.ajp.2009.08091307 19411370 PMC2885958

[B108] Rogers T. D. Dickson P. E. Heck D. H. Goldowitz D. Mittleman G. Blaha C. D. (2011). Connecting the dots of the cerebro-cerebellar role in cognitive function: neuronal pathways for cerebellar modulation of dopamine release in the prefrontal cortex. Synapse 65, 1204–1212 10.1002/syn.20960 21638338 PMC3854794

[B109] Rogers T. D. Dickson P. E. McKimm E. Heck D. H. Goldowitz D. Blaha C. D. (2013). Reorganization of circuits underlying cerebellar modulation of prefrontal cortical dopamine in mouse models of autism spectrum disorder. Cerebellum 12, 547–556 10.1007/s12311-013-0462-2 23436049 PMC3854915

[B110] Saalmann Y. B. Kastner S. (2011). Cognitive and perceptual functions of the visual thalamus. Neuron 71, 209–223 10.1016/j.neuron.2011.06.027 21791281 PMC3148184

[B111] Salgado-Pineda P. Landin-Romero R. Fakra E. Delaveau P. Amann B. L. Blin O. (2014). Structural abnormalities in schizophrenia: further evidence on the key role of the anterior cingulate cortex. Neuropsychobiology 69, 52–58 10.1159/000356972 24457222

[B112] Schmahmann J. D. (1998). Dysmetria of thought: clinical consequences of cerebellar dysfunction on cognition and affect. Trends Cogn. Sci. 2, 362–371 10.1016/s1364-6613(98)01218-2 21227233

[B113] Schmahmann J. D. (2004). Disorders of the cerebellum: ataxia, dysmetria of thought and the cerebellar cognitive affective syndrome. J. Neuropsychiatry Clin. Neurosci. 16, 367–378 10.1176/appi.neuropsych.16.3.367 15377747

[B114] Schmahmann J. D. (2010). The role of the cerebellum in cognition and emotion: personal reflections since 1982 on the dysmetria of thought hypothesis and its historical evolution from theory to therapy. Neuropsychol. Rev. 20, 236–260 10.1007/s11065-010-9142-x 20821056

[B115] Schulz R. Wessel M. J. Zimerman M. Timmerman J. Gerloff C. Hummel F. C. (2014). White matter integrity of specific dentato-thalamo-cortical pathways is associated with learning gains in precise movement timing. Cereb. Cortex [Epub ahead of print]. 10.1093/cercor/bht356 24443417

[B116] Schutter D. J. L. G. van Honk J. d’ Alfonso A. A. L. Peper J. S. Panksepp J. (2003). High frequency repetitive transcranial magnetic over the medial cerebellum induces a shift in the prefrontal electroencephalography gamma spectrum: a pilot study in humans. Neurosci. Lett. 336, 73–76 10.1016/s0304-3940(02)01077-7 12499043

[B117] Seeman P. (1987). Dopamine receptors and the dopamine hypothesis of schizophrenia. Synapse 1, 133–152 10.1002/syn.890010203 2905529

[B118] Shevelkin A. V. Ihenatu C. Pletnikov M. V. (2014). Pre-clinical models of neurodevelopmental disorders: focus on the cerebellum. Rev. Neurosci. 25, 177–194 10.1515/revneuro-2013-0049 24523305 PMC4052755

[B119] Siegel J. J. Kalmbach B. Chitwood R. A. Mauk M. D. (2012). Persistent activity in a cortical-to-subcortical circuit: bridging the temporal gap in trace eyelid conditioning. J. Neurophysiol. 107, 50–64 10.1152/jn.00689.2011 21957220 PMC3349685

[B120] Sivaswamy L. Kumar A. Rajan D. Behen M. Muzik O. Chugani D. (2010). A diffusion tensor imaging study of the cerebellar pathways in children with autism spectrum disorder. J. Child Neurol. 25, 1223–1231 10.1177/0883073809358765 20179000

[B121] Snider R. S. Maiti A. Snider S. R. (1976). Cerebellar pathways to ventral midbrain and nigra. Exp. Neurol. 53, 714–728 10.1016/0014-4886(76)90150-3 1001395

[B122] Stahl S. M. Buckley P. F. (2007). Negative symptoms of schizophrenia: a problem that will not go away. Acta Psychiatr. Scand. 115, 4–11 10.1111/j.1600-0447.2006.00947.x 17201860

[B123] Stoodley C. J. Schmahmann J. D. (2009). The cerebellum and language: evidence from patients with cerebellar degeneration. Brain Lang. 110, 149–153 10.1016/j.bandl.2009.07.006 19664816

[B124] Strick P. L. Dum R. P. Fiez J. A. (2009). Cerebellum and nonmotor function. Annu. Rev. Neurosci. 32, 413–434 10.1146/annurev.neuro.31.060407.125606 19555291

[B125] Sudarov A. (2013). Defining the role of cerebellar Purkinje cells in autism spectrum disorders. Cerebellum 12, 950–955 10.1007/s12311-013-0490-y 23703312 PMC3795842

[B126] Takayanagi M. Wentz J. Takayanagi Y. Schretlen D. J. Ceyhan E. Wang L. (2013). Reduced anterior cingulate gray matter volume and thickness in subjects with deficit schizophrenia. Schizophr. Res. 150, 484–490 10.1016/j.schres.2013.07.036 24035178 PMC4076020

[B127] Tomlinson S. P. Davis N. J. Bracewell R. M. (2013). Brain stimulation studies of non-motor cerebellar function: a systematic review. Neurosci. Biobehav. Rev. 37, 766–789 10.1016/j.neubiorev.2013.03.001 23500608

[B128] Tsubota T. Ohashi Y. Tamura K. Sato A. Miyashita Y. (2011). Optogenetic manipulation of cerebellar purkinje cell activity in vivo. PLoS One 6:e22400 10.1371/journal.pone.0022400 21850224 PMC3151259

[B129] Uylings H. B. M. Groenewegen H. J. Kolb B. (2003). Do rats have a prefrontal cortex? Behav. Brain Res. 146, 3–17 10.1016/j.bbr.2003.09.028 14643455

[B130] Vilensky J. A. Van Hoesen G. W. (1981). Corticopontine projections from the cingulate cortex in the rhesus monkey. Brain Res. 205, 391–395 10.1016/0006-8993(81)90348-6 7470872

[B131] Vogt B. A. Hof P. R. Zilles K. Vogt L. J. Herold C. Palomero-Gallagher N. (2013). Cingulate area 32 homologies in mouse, rat, macaque and human: cytoarchitecture and receptor architecture. J. Comp. Neurol. 521, 4189–4204 10.1002/cne.23409 23840027

[B132] Vogt B. A. Paxinos G. (2014). Cytoarchitecture of mouse and rat cingulate cortex with human homologies. Brain Struct. Funct. 219, 185–192 10.1007/s00429-012-0493-3 23229151

[B133] Volz H.-P. Nenadic I. Gaser C. Rammsayer T. Häger F. Sauer H. (2001). Time estimation in schizophrenia: an fMRI study at adjusted levels of difficulty. Neuroreport 12, 313–316 10.1097/00001756-200102120-00026 11209941

[B134] Walker E. Shaye J. (1982). Familial schizophrenia. A predictor of neuromotor and attentional abnormalities in schizophrenia. Arch. Gen. Psychiatry 39, 1153–1156 10.1001/archpsyc.1982.04290100027005 7125845

[B135] Wassink T. H. Andreasen N. C. Nopoulos P. Flaum M. (1999). Cerebellar morphology as a predictor of symptom and psychosocial outcome in schizophrenia. Biol. Psychiatry 45, 41–48 10.1016/s0006-3223(98)00175-9 9894574

[B136] Watson T. C. Becker N. Apps R. Jones M. W. (2014). Back to front: cerebellar connections and interactions with the prefrontal cortex. Front. Syst. Neurosci. 8:4 10.3389/fnsys.2014.00004 24550789 PMC3912388

[B137] Watson T. C. Jones M. W. Apps R. (2009). Electrophysiological mapping of novel prefrontal - cerebellar pathways. Front. Integr. Neurosci. 3:18 10.3389/neuro.07.018.2009 19738932 PMC2737490

[B138] Weinberger D. R. Berman K. F. Zec R. F. (1986). Physiologic dysfunction of dorsolateral prefrontal cortex in schizophrenia. I. Regional cerebral blood flow evidence. Arch. Gen. Psychiatry 43, 114–124 10.1001/archpsyc.1986.01800020020004 3947207

[B139] Weinberger D. R. Kleinman J. E. Luchins D. J. Bigelow L. B. Wyatt R. J. (1980). Cerebellar pathology in schizophrenia: a controlled postmortem study. Am. J. Psychiatry 137, 359–361 7356066 10.1176/ajp.137.3.359

[B140] Weiss C. Disterhoft J. F. (1996). Eyeblink conditioning, motor control and the analysis of limbic-cerebellar interactions. Behav. Brain Sci. 19, 479–481 10.1017/s0140525x00081929

[B141] White T. Nelson M. Lim K. O. (2008). Diffusion tensor imaging in psychiatric disorders. Top. Magn. Reson. Imaging 19, 97–109 10.1097/rmr.0b013e3181809f1e 19363432

[B142] Williams S. M. Goldman-Rakic P. S. (1998). Widespread origin of the primate mesofrontal dopamine system. Cereb. Cortex 8, 321–345 10.1093/cercor/8.4.321 9651129

[B143] Wiser A. K. Andreasen N. C. O’Leary D. S. Watkins G. L. Boles Ponto L. L. Hichwa R. D. (1998). Dysfunctional cortico-cerebellar circuits cause “cognitive dysmetria” in schizophrenia. Neuroreport 9, 1895–1899 10.1097/00001756-199806010-00042 9665622

[B144] Wu T. Hallett M. (2013). The cerebellum in Parkinson’s disease. Brain 136, 696–709 10.1093/brain/aws360 23404337 PMC7273201

[B145] Yücel M. Pantelis C. Stuart G. W. Wood S. J. Maruff P. Velakoulis D. (2002). Anterior cingulate activation during Stroop task performance: a PET to MRI coregistration study of individual patients with schizophrenia. Am. J. Psychiatry 159, 251–254 10.1176/appi.ajp.159.2.251 11823267

